# Old habits die hard; does early urinary catheter removal affect kidney size, bacteriuria and UTI after renal transplantation?

**DOI:** 10.15171/jrip.2017.08

**Published:** 2016-11-20

**Authors:** Roghayeh Akbari, Sedigheh Rahmani Firouzi, Abazar Akbarzadeh-Pasha

**Affiliations:** ^1^Clinical Research Development Unit, Ayatollah Rohani Hospital, Babol University of Medical Sciences, Babol, Iran; ^2^Babol University of Medical Sciences, Babol, Iran

**Keywords:** Kidney transplantation, Bacteriuria, Urinary tract infection

## Abstract

**Introduction:** Renal transplantation is the treatment of choice in chronic renal failure patients.

**Objectives:** The purpose of this study was to evaluate the impact of urinary catheter removal time on transplanted kidney size and incidence of asymptomatic bacteriuria and urinary tract infections (UTIs).

**Patients and Methods:** This retrospective cohort study evaluated the clinical outcomes of 109 consecutive live donor renal transplant recipients from December 2011 to July 2014. Routine ultrasound examinations were performed on donor’s kidney prior to operation and one month later. Kidney volume was calculated. UTI and bacteriuria were evaluated one month later. Patients were divided into two groups based on time of Foley catheter removal (before and after fifth day posttransplantation).

**Results:** In this study 74 males (67.9%) and 35 females (32.1%) were evaluated. Sixty-six patients (57.92%) were in group 1. None of the patients with positive urine culture had UTI but bacteriuria occurred in all of them (21.1%). Bacteriuria time after transplantation and catheter removal was significantly later in group 1 and it was not different in female group but they were later in male group. The mean renal volume increase was positively correlated to renal transplant recipient and donor’s age and donor’s body mass index (BMI) (*P*<0.05).

**Conclusion:** This study showed that the time of catheter removal after kidney transplantation does not affect incidence of UTI but increases the probability of bacteria in men whose catheter was removed within 5 days after transplantation. We also found that the renal volume change is not associated with catheter removal time and bacteriuria.

**Implication for health policy/practice/research/medical education:**

In a study on 109 patients, we found the time of catheter removal after kidney transplantation does not affect incidence of UTI but increases the probability of bacteria in men whose catheter was removed within 5 days after transplantation. We also found that the renal volume change is not associated with catheter removal time and bacteriuria.

## Introduction

Renal transplantation is the treatment of choice in chronic renal failure patients and treatment outcomes varies in different centers (1). Urinary tract infection (UTI) is the most common infection following renal transplantation with a prevalence of 35% to 79% and accounts for about 40%-50% of all infectious complications (2,3). It is associated with the development of acute cellular rejection, impaired allograft function, allograft loss, and death. Morbidity and mortality from UTI can be caused by recurrent and/or severe sepsis (4). The importance of this concern is further underscored by the fact that about 60% of bacteremia among renal transplant recipients originates from urinary tract, so its prevention is important (5). Risk factors for UTI include old age, female gender, cadaveric donor, UTI before transplantation, prolonged hemodialysis period, polycystic renal disease, diabetes mellitus, prolonged postoperative bladder catheterization, reflux kidney disease immunosuppression, allograft trauma, and technical complications associated with urethral anastomosis (6).

All aspects of pre- and postoperative care of renal transplant recipients need to be evaluated to ensure optimal patient safety and clinical outcomes. Catheterization of the urinary bladder during renal transplantation is essential because postoperative urine output measurement with Foley catheterization is essential (7). Its benefits include preparation of the bladder for anastomosis and monitoring subsequent urinary output (8). The optimal time to remove the catheter postoperatively is controversial. The trend in most institutions is to err on the side of leaving the catheter in for a longer period of time, to ensure consistent urine output (7). Some recommend catheter removal on postoperative day 5 (9). The risks of prolonged Foley catheterization are manifold. It increases the risk of catheter-associated UTI especially in newly transplanted patients receiving immunosuppression. It also decreases patient mobility which increases the risk of venous thromboembolism, pneumonia, and hospital length of stay. It is suggested to remove the catheter on postoperative day 1 in live donor renal transplant recipients to prevent its complications (7).

Bladder drainage with Foley catheterization may allow the probable leak to heal before becoming clinically manifest. Abnormalities of kidney size occur in many renal diseases. The determination of normal renal size in any population is important in the diagnosis, treatment and prognosis of renal disease (10). Also, kidney size using either renal length, volume, and cortical thickness as a unit of measurement represent renal function and are used frequently as the basis for making clinical decisions in the evaluation and follow up of kidney transplant patients (11). The most accurate of these parameters is renal volume, as the shape of kidney varies considerably. Serial measurements also can provide information regarding disease progression or stability (12).

Because therapeutic decisions frequently are based on the results of these measurements, accurate measurement of renal length and volume are of increasing importance (12).

## Objectives

The purpose of this study was to evaluate the impact of urinary catheter removal time on transplanted kidney size changes and incidence of asymptomatic bacteriuria and UTIs one month following the operation.

## Patients and Methods

In this retrospective cohort study we reviewed the clinical outcomes of 127 consecutive live donor renal transplant recipients from December 2011 until July 2014. In case of neurogenic bladder, history of recurrent UTI, history of UTI one month prior to renal transplantation, history of frequent catheterizations, abnormal urogenital system, history of vesicoureteral reflux, loss of kidney transplant due to antibody mediated rejection and frequent catheter manipulation after transplantation, patients were excluded from the study. Finally, 109 patients remained.

### Kidney transplantation

All the surgical procedures were performed by the same surgeon and surgery team in the same center from live donors. The kidney graft was placed in iliac cavity close to the previously dissected external iliac vessels. Anastomosis of renal vein to the common or external iliac vein and renal artery to internal or external iliac artery were performed. In all patients a silicon double J stent or Nelaton catheter (Ch/Fr 8) was inserted at operation room as urethral stent. The time of urethral stent removal was similar in all patients. The initial dose of immunosuppressant was given immediately after the transplantation. Subsequently, the patients were kept on maintenance dose and the immunosuppressant protocol were the same in all patients.

### Transplanted kidney size

Routine ultrasound examinations were performed on donor’s kidney prior to operation and by one radiologist. Upper and lower poles of kidney were identified and their distance was used as renal length (the distance between the highest and lowest levels of kidney). The anteroposterior (AP) diameter of the kidney in the middle one third was considered as renal cortical thickness.

The renal width represented the average measurement from the widths at the hilum and 1 cm above and 1 cm below the hilum. These widths were perpendicular to the AP diameter. Thus, the volume was calculated according to the equation for an ellipsoid (13): 0.523 × *L* × *W* × *AP*. Here, *L* is the maximum bipolar diameter (BPD); *W* is the renal width and *AP* is the anteroposterior diameter.

Ultrasound examination was performed one month after the operation on transplanted kidney and all diameters were remeasured and the volume was recalculated.

### Bacterial identification

UTI and bacteriuria were evaluated one month after the transplantation. Qualitative urine cultures were performed on blood agar and McConkey agar (Himedia, India) and for grown bacteria, biochemical identification tests and colony count were done as per standard protocol. The first urine sample for urine analysis and culture was obtained within 12 hours following catheter removal and then, routine urine cultures were performed on Saturdays and Tuesdays by the patients from midstream urine. During the catheterization, in case of urinary signs, a sample for urine analysis and culture was obtained. A positive result for UTI was considered when the bacterial counts were recorded more than 10^5^ bacterial colony-forming unit per milliliter (CFU/mL) on urine culture with local urinary symptoms. Local urinary symptoms included dysuria, frequency, or urgency, suprapubic tenderness and fever (2).

Asymptomatic bacteriuria was defined by the presence of more than 10^5^CFU/mL of urine on urine culture with no local or systemic symptoms of UTI (4).

Antibiogram was performed for all urine cultures and the pathogen susceptibility was obtained. All urine cultures were performed in one laboratory. Antibiotic prophylaxis with cefazolin was done and after the catheter removal, amikacin was prescribed and cotrimoxazol was continued. Patients with UTI were treated with meropenem.

### Catheterization

For all kidney graft recipients, a Foley catheter was inserted in the operation room prior to renal transplantation which was removed within 5 days of transplantation. Prolonged use of Foley catheter was defined as the use of a Foley catheter beyond day 5 after transplantation. The physician removed the catheter randomly whenever decided and without being informed of the study protocol. Then, the patients were divided into two groups. For the first group, the catheter was removed before 5 days postoperation and for the second group this time was after the fifth day.

Finally, data including age, gender, height, weight, history of diabetes mellitus and thymoglobulin prescription for the renal transplant recipient were recorded. BMI was calculated using weight divided by the square of the body height (kg/m^2^). Body surface area was calculated using the formula (14): 71.84 × height (m)^0.725^× weight (kg)^0.425^.

### Ethical issues

The research followed the tenets of the Declaration of Helsinki and informed consent was obtained. The research was approved by the ethical committee of Babol University of Medical Sciences (#923649, 2013).

### Statistical analysis

Data was analyzed by SPSS version 17.0. A descriptive analysis was performed, defining the baseline population characteristics and exploring the main results in the cohort. Chi-square, *t* test and logistic regression tests were used to analyze different factors in both groups. A *P* value less than 0.05 was considered being statistically significant.

## Results

This study consisted of 109 patients, 74 males (67.9%) and 35 females (32.1%) with the mean age of 42.0±13.05 years for donors and 28.17±4.20 years for renal transplant recipients. The most common reasons for end-stage renal disease were unknown (33%) and hypertension (30.3%). Sixty-six patients (57.92%) were in group 1 and 46 patients (41.07%) were in group 2.

Donor and recipient gender, thymoglobulin prescription, recipient diabetes and right and left transplanted kidney sizes were not in both groups (Table 1). Also, the mean age, BMI and body surface area (BSA) were not statistically different between two groups (Table 2).

**Table 1:** Qualitative variables in both groups

	**Group 1** **(n=63)**	**Group 2** **(n=46)**	***P*** ** value**
Donor gender			0.610
Male	47 (74.6%)	37 (80.4%)	
Female	16 (25.4%)	9 (19.6%)	
Recipient gender			0.474
Male	44 (69.8%)	30 (65.2%)	
Female	19 (30.2%)	16 (34.8%)	
Thymoglobulin prescription	6 (9.5%)	9 (20.0%)	0.121
Recipient diabetes	9 (14.3%)	8 (17.4%)	0.659
Transplanted kidney			0.680
Right	10 (15.9%)	6 (12.0%)	
Left	53 (84.1%)	40 (87.0%)	

**Table 2:** Quantitative variables and the mean time to bacteriuria in both groups

	**Group 1** **(n=63)**	**Group 2 (n=46)**	**P value**
Age (year)			
Donor	27.92±4.16	28.50±4.32	0.480
Recipient	42.52±12.7	41.28±13.62	0.620
BMI (kg/m^2^)			
Donor	24.91±3.51	24.64±3.73	0.703
Recipient	24.04±3.78	24.93±4.76	0.280
BSA (m^2^)			
Donor	650.54±59.76	652.82±68.87	0.854
Recipient	608.86±75.28	618.11±81.58	0.542
Bacteriuria time post-donation (day)	2.37±5.21	0.65±2.00	0.020
Bacteriuria time after catheter removal (day)	1.33±3.95	0.09±0.354	0.016
Renal length (cm)			
Donor	11.81±0.76	11.86±0.78	0.760
Recipient	115.76±13.61	114.52±10.58	0.608
Renal width (cm)			
Donor	4.75±0.66	4.84±0.70	0.481
Recipient	5.05±0.99	5.08±0.88	0.862
AP diameter			
Donor	5.80±0.92	5.90±1.26	0.672
Recipient	6.21±1.10	6.00±0.79	0.280
Renal volume (cm^3^)			
Donor	173.32±49.94	181.21±64.08	0.470
Recipient	168.20±79.95	188.87±66.43	0.520

Abbreviations: BMI, body mass index; AP, anteroposterior; BSA, body surface area

None of the patients with positive urine culture had UTI symptoms but bacteriuria occurred in all of them (21.1%). The mean catheter removal time in group 1 was 4.59±0.687 days and 7.67±2.522 days in group 2. As shown in Table 2, kidney size of donor and recipients were not different. Also, the kidney sizes were not statistically different based on gender (*P*>0.05).

Bacteriuria time after transplantation and catheter removal was statistically different in both groups and was later in group one which removed the catheter sooner. After considering gender, bacteriuria time after transplantation and catheter removal were not different in female group (*P*=0.462 and *P*=0.098, respectively) but they were later in male group (*P*=0.014 and *P*=0.049, respectively). Of 23 cases (21.1%) with bacteriuria, 17 (73.9%) were in group 1 and 6 ones (26.1%) were in group 2 (*P*=0.078). Five (21.74%) of them occurred during catheterization and 18 (78.26%) occurred after catheter removal. Of these, the mean renal volume increased 16.27 cm^3^ which was not different from patients without bacteriuria (17.96 cm^3^ increase, *P*=0.922).

Logistic regression for the association between bacteriuria and age, gender, thymoglobulin prescription, diabetes mellitus and catheter removal time showed that only gender (odds ratio [OR]=2.99, 95% CI=1.13-8.91, *P*=0.031) and catheter removal time (OR=0.30, 95% CI=0.09-0.92, *P*=0.039) were significantly associated with bacteriuria so that in women, it was three times higher than men. The most common isolated bacteria were gram negative bacilli as follows: *Escherichia coli* (15.6%), *Enterobacter* spp. (2.7%), *Citrobacter* (1.8%) and *Pseudomonas aeruginosa* (0.9%) and the antibiotic of choice was amikacin.

Male gender and bacteriuria after catheter removal were significantly associated (*P*=0.021) meaning that removing catheter before the fifthday of transplantation leads to higher rates of UTI and bacteriuria after catheter removal. Also, female gender and bacteriuria after catheter removal were not associated (*P*=0.728) meaning that removing catheter before the fifthday of transplantation does not increase UTI and bacteriuria after catheter removal.

One month after transplantation, the mean renal volume increase in group 1 was 78.35±24.88 cm^3^and it was 65.26±7.63 cm^3^in group 2. Although this volume elevation was higher in group 1, but it was not statistically significant (*P*=0.227). We also found that the difference of renal volume was positively correlated to renal transplant recipient and donor’s age and donor’s BMI (*P*=0.000, *P*=0.008 and *P*=0.037, respectively). With increasing BMI and age of recipient, the renal volume increased after one month but increasing donor’s age leaded to renal volume decrease after one month.

The mean renal volume increased in both male and females (76.99±23.08 cm^3^and 64.29±6.01 cm^3^, respectively, *P*=0.258).

## Discussion

One of the main complications after renal transplantation with serious consequences is UTI (15). These patients are also at high risk for developing bacteriuria which is dangerous for patient and the transplanted kidney (16). Treatment of patients with severe renal failure has been enhanced, especially with improvement in surgical procedures and pharmaceutical management. Nevertheless, due to immunosuppressive treatment, these patients are capable to develop postoperative infections and UTI (17). To our knowledge, this is the first study to evaluate the association between kidney size and volume with UTI and bacteriuria. Previous studies have evaluated the effect of donor kidney size on recipient outcomes.

In this study bacteriuria occurred in 21.1% of the patients one month after transplantation and bacteriuria time after transplantation and catheter removal was later in group 1 which removed the catheter sooner. The study of Khosravi et al demonstrated the UTI rate of 33.56% among renal transplant recipients (5). In the studies of Rivera-Sanchez et al and Kanisauskaite et al, 33.3% and 37% of patients developed UTI (15,18). Besides, it was similar to the studies of Lee et al (21%) and Glazer et al (16.0%) which removed the catheter before 6 days of transplantation (2,8) but the reported rate of UTI was 53.69% in Saudi Arabia (19), 77% in Pakistan (20), and 63% in Japan (21), which was higher than our study. Occurrence of UTI depends on many factors such as age, female gender, co-morbidity, type and amount of immunosuppression, prolonged use of Foley catheter, urological instrumentation and/or length of follow up period after kidney transplantation (3). The main reason for lower rate of bacteriuria in our study may be earlier catheter removal in comparison with other studies. Another reason may be better surgical procedures and pharmaceutical management (amikacin prophylaxis) since the time they conducted the study. Also, in previous studies, patients with structural abnormalities (reflux uropathy, bladder outlet obstruction) were studied and developed UTI but we excluded them from the study (20).

In this study, the most common isolated bacteria were* E. coli*, *Enterobacter* spp*.*, *Citrobacter* and *P. aeruginosa*. In the study of Iqbal et al, the principally isolated agents were *E.coli* and *Pseudomonas* followed by *Pseudomonas, Klebsiella,* and *Morganella morgagn* (20). It should be noted that the most frequent organisms demonstrated in renal transplant recipients are *E. coli* and other gram negative bacteria, such as *Klebsiella* spp*., Enterobector* spp*.,* and *P. aeruginosa* (22). The study of Sharma et al demonstrated *E. coli* as a common isolate followed by *Staphylococcus*, *Enterococcus* and *Pseudomonas* (23). In the study of Khosravi et al, *E. coli* was isolated in 43.53% of cases, followed by *Enterobacter* spp*.*, and *P. aeruginosa* (5).

This study showed that early catheter removal causes less bacteriuria than later one. In the study of Iqbal et al posttransplant hospital stay and delay in removal of Foleys catheter were statistically significant factors causing UTI in transplant population (20). The study by Dantas et al also demonstrated the duration of urinary bladder catheterization as main risk factor for UTIs in posttransplant patients (24). Siskind et al found that removal of Foley catheters in live donor renal transplant recipients on postoperative day 1 decreases the risk of catheter-associated UTIs and length of hospital stay (7). It also improves patient comfort, and promotes early postoperative ambulation (8).

In the current study, the prevalence of bacteriuria in women was higher in both groups. In accordance to our study, a statistically significant higher incidence of UTI was reported by other similar studies in female patients but no significant difference was observed between gender of the patients and the incidence of UTI in the study of Khosravi et al (5,15,16,19).

In accordance with other reports, we found that removing catheter before the fifth day of transplantation leads to higher rates of UTI and bacteriuria in men but not in women. Female patients have been reported to have a higher risk of developing UTI than men after renal transplantation (25). We did not encounter any side effects and this approach is probably more comfortable for patients. Except early catheter removal, other risk factors for UTIs like female gender, previous UTI and type II diabetes are not modifiable. So, early catheter removal appears to be an effective method to decrease UTI in these patients (8).

In our study, the mean renal volume increase in both male and females one month after transplantation but it was not associated to bacteriuria. There was a significant enlargement of the kidney size in the study of Khosroshahi et al (26). After renal transplantation, patients are routinely treated with immunosuppressive drugs to minimize the risk of allograft rejection. There are no distinguished clinical features to differentiate immunosuppressive drugs’ nephrotoxicity from acute rejection. The histology can be nonspecific sometimes, with evidence of both toxicity and rejection in a single biopsy (27). A change in kidney dimensions from one examination to the next may be an important indicator of the presence or progression of disease (12). Renal length and volume also are important clinical parameters in the evaluation and follow-up of kidney transplant recipients (11).

We observed that one month after transplantation, the mean renal volume increase was higher in patients whose catheter was removed sooner but it was not statistically significant. Kidney volume provides an adequate estimate of nephron number, which, if low, increases the risk of hypertension and renal failure (28). Most of the studies have shown strong correlations between kidney volume and post-transplantation outcomes in living donor transplantation (29-31).

This study revealed that with increasing BMI and age of the recipients, the renal volume increased after one month but increasing donor’s age decreased renal volume after one month. In the study of Cheong et al in both men and women, renal volumes decreased with age but this trend was not statistically significance (12).

## Conclusion

This study showed that the time of catheter removal after kidney transplantation does not affect incidence of bacteriuria but increases the probability of bacteriuria in men whose catheter was removed within 5 days after transplantation. We also found that the renal volume change is not associated with catheter removal time and bacteriuria.

## Limitations of the study

We had some limitations. First, its short follow-up time limited us to state with certainty that donated kidney volume predicts long-term graft survival in living renal transplantation after this time (15,19). Previous studies have shown that kidney function one year after transplantation is predictive of long-term outcome (32). We used a retrospective cohort study design to identify independent risk factors for UTI and subsequent bacteremia. There was a significant gender disproportion in this study. It is suggested to evaluate risk factors for UTI including pre-transplant urine volume, post void residual volume, and prostate volume. Longer follow-up periods will be needed to determine the real impact of kidney size on graft survival is suggested.

## Acknowledgements

It is our pleasure to thank all the laboratory staff of Shahid Beheshti hospital, Babol University of Medical Sciences, Iran. We would also thank Mrs. Fatemeh Hosseinzadeh (Infectious Diseases Research Center with focus on Nosocomial Infections, Mazandaran University of Medical Sciences) for editing the manuscript.

## Authors’ contribution

RA, and AAP participated in all experiments, coordinated the data-analysis and contributed to the writing of the manuscript. SRF coordinated the acquisition of data. RF and AAP designed the research plan and organized the study. RA prepared the final manuscript.

## Conflicts of interest

The authors declare no conflict of interest.

## Ethical considerations

Ethical issues (including plagiarism, data fabrication, double publication) have been completely observed by the authors.

## Funding/Support

This article is extracted from general physician thesis of SRF. This study was supported by a grant from Babol University of Medical Sciences (#923649, 2013).
